# Neuroendocrine tumor of the ampulla of Vater with distant cystic lymph node metastasis: a case report

**DOI:** 10.1186/s40792-016-0202-1

**Published:** 2016-07-25

**Authors:** Mariko Tsukagoshi, Yasuo Hosouchi, Kenichiro Araki, Yasushi Mochida, Ryusuke Aihara, Ken Shirabe, Hiroyuki Kuwano

**Affiliations:** 1 Department of General Surgical Science, Gunma University Graduate School of Medicine, 3-39-22 Showa-machi, Maebashi, Gunma 371-8511 Japan; 2 Department of Surgery and Laparoscopic Surgery, Gunma Prefecture Saiseikai-Maebashi Hospital, 564-1 Kamishinden-machi, Maebashi, Gunma 371-0821 Japan; 3 Department of Hepatobiliary and Pancreatic Surgery, Gunma University Graduate School of Medicine, 3-39-22 Showa-machi, Maebashi, Gunma 371-8511 Japan

**Keywords:** Neuroendocrine tumor, Lymph node metastases, Ampulla of Vater, Surgical resection

## Abstract

**Background:**

Neuroendocrine tumors (NETs) of the ampulla of Vater are rare and difficult to diagnose. We report a rare case of a small NET of the ampulla of Vater with metastasis to distant lymph nodes.

**Case presentation:**

The patient was a 54-year-old man complaining of epigastric pain and melena. Upper gastrointestinal endoscopy revealed a bulging papilla with active bleeding, which was diagnosed as a well-differentiated NET of the ampulla of Vater. An approximately 10-mm hypervascular tumor at the ampulla of Vater and a 41-mm cyst adjacent to the wall of the jejunum were revealed by abdominal computed tomography. We performed pylorus-preserving pancreaticoduodenectomy with lymph node dissection. Macroscopic examination revealed a 9-mm tumor of the ampulla of Vater and a 52-mm cyst adjacent to the wall of the jejunum. Histological examination revealed that the cyst was a lymph node metastasis. The final diagnosis was non-functional NET G1 of the ampulla of Vater, designated T1N1M0 stage IIIB. Postoperatively, the patient underwent no treatment and has had no recurrence for 4 years.

**Conclusions:**

This case demonstrates that sporadic NETs of Vater’s papilla have aggressive metastatic potential even with a small primary lesion, and radical resection with lymphadenectomy is recommended for all cases.

## Background

Neuroendocrine tumors (NETs) of the ampulla of Vater are rare [1, 2]. A total of 139 patients with NETs of the ampulla were identified from the Surveillance, Epidemiology, and End Results Program of the National Cancer Institute between 1973 and 2006 [3]. The incidence of NETs has been rising due to an improvement in diagnostic techniques, and the mortality rate has also been increasing in recent years [4, 5]. There are still many issues regarding diagnosis and treatment.

There is no curative treatment for NETs, except surgical resection. In general, a tumor size greater than 2 cm in diameter, invasion of the muscularis propria, and presence of mitotic figures are correlated with metastasis of NETs [6]. Therefore, surgery has been recommended for tumors larger than 2 cm.

In the cases of duodenal NETs, tumors smaller than 2 cm have limited metastatic potential [7, 8]. However, unlike NETs in other areas, it has been demonstrated that tumor size has no correlation with metastatic potential in NETs of the ampulla of Vater [1, 9]. Thus, the best therapeutic approach for ampullary NETs remains controversial.

Herein, we report our experience with a rare case of 9-mm ampullary NET with lymph node metastasis and review the current literatures on the topic.

## Case presentation

**Fig. 1:**
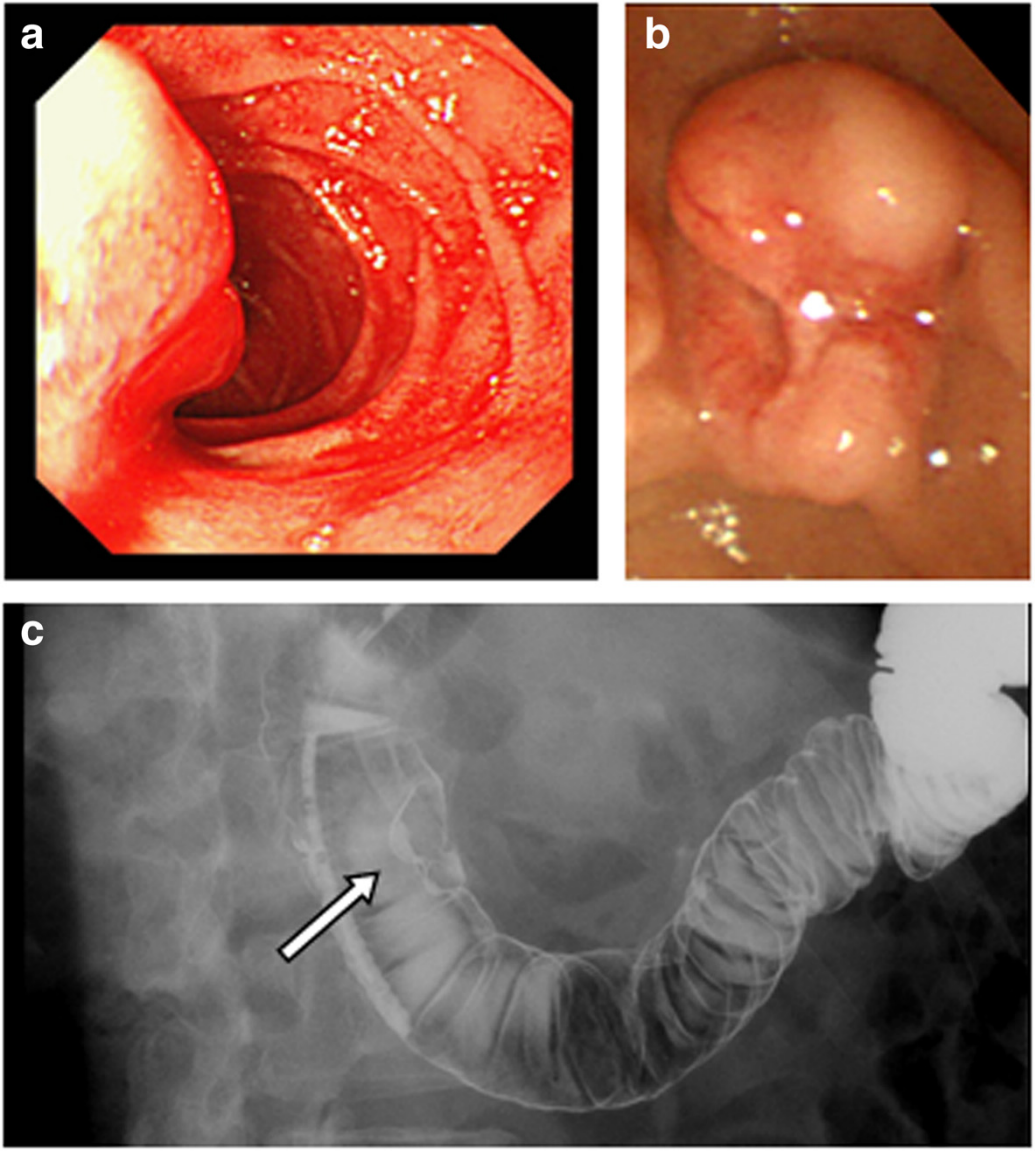
Upper gastrointestinal endoscopy shows a bulging papilla with bleeding on day 1 (**a**) and shows an approximately 10-mm mass of the ampulla of Vater with a superficial ulcer on day 7 (**b**). Upper gastrointestinal series shows an ampullary mass (*arrow*) (**c**)

**Fig. 2:**
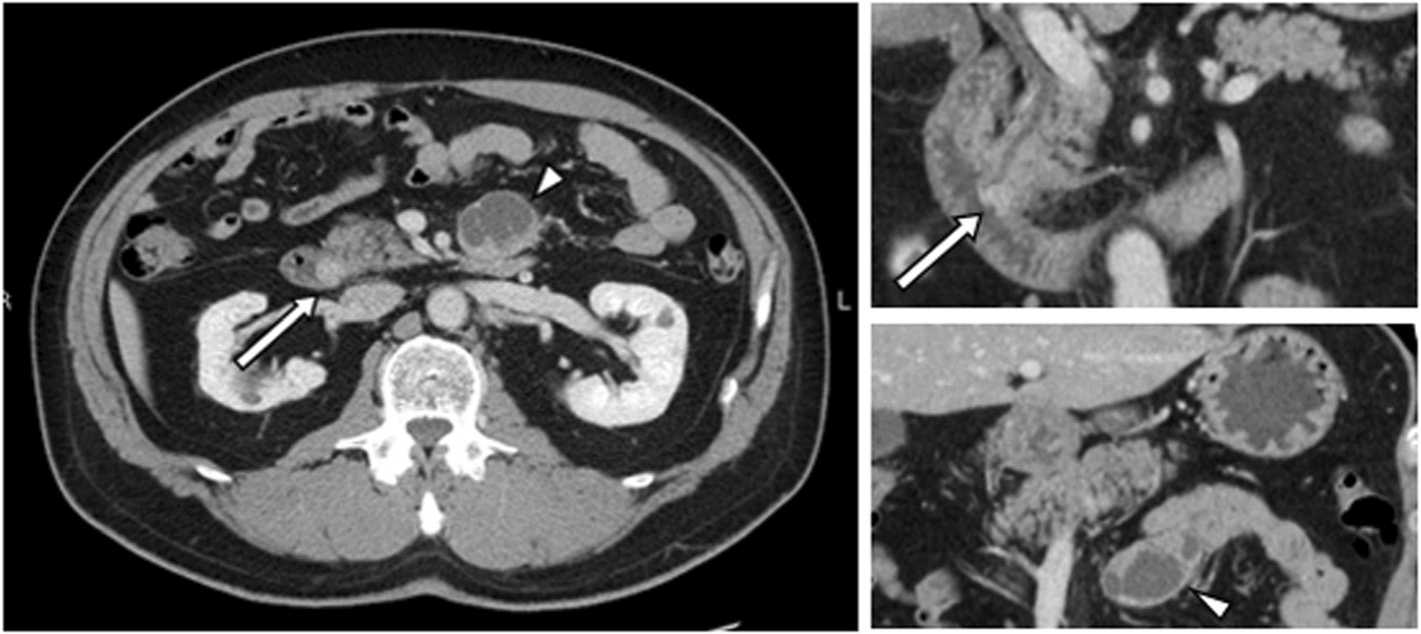
Enhanced abdominal computed tomography scan shows a 10-mm hypervascular tumor at the ampulla of Vater (*arrow*) and a 41-mm multilocular cyst adjacent to the wall of the jejunum near the ligament of Treitz (*arrowhead*)

A 54-year-old man presented with a history of epigastric pain and melena over the last few days. The patient did not have hormone-related symptoms. He had received treatment for a myocardial infarction at the age of 51 with an antiplatelet therapy; therefore, we considered that there was a possibility of tumor bleeding by antiplatelet therapy. Blood biochemical examinations indicated slight anemia. Serum carbohydrate antigen 19-9 and carcinoembryonic antigen (CEA) levels were not elevated. The 24-h urinary 5-hydroxyindoleacetic acid (5-HIAA) level was 3.2 mg. Upper gastrointestinal endoscopy showed a bulging papilla with bleeding (Fig. 1a). A biopsy was not performed at that time because of the active bleeding. Endoscopic examination on day 7 revealed an approximately 10-mm mass of the ampulla of Vater with a superficial ulcer (Fig. 1b). Pathological findings of the endoscopic biopsy of the ampulla revealed a well-differentiated NET. Immunohistochemically, the tumor stained positive for CD56, chromogranin A, and synaptophysin. An upper gastrointestinal series showed an ampullary mass without any obvious lesions of the jejunum (Fig. 1c). An enhanced abdominal computed tomography (CT) scan revealed a 10-mm hypervascular tumor at the ampulla of Vater and a 41-mm multilocular cyst adjacent to the wall of the jejunum near the ligament of Treitz (Fig. 2). The wall of the multilocular cyst showed the same enhancement patterns with the tumor. The patient underwent magnetic resonance imaging that was negative for visceral metastasis. About the cyst, the preoperative definitive diagnosis was not provided.

**Fig. 3:**
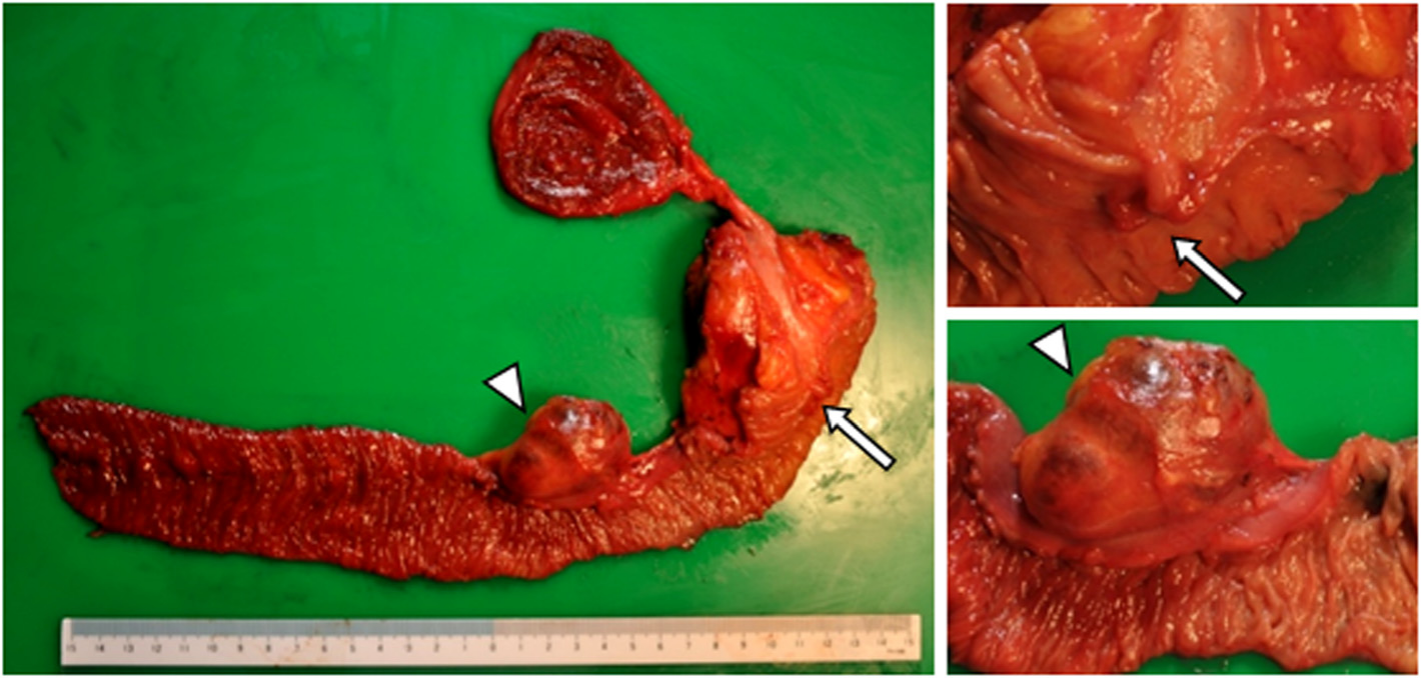
Pathological findings show a 9-mm white solid ampullary tumor (*arrow*) and a 52-mm cyst adjacent to the wall of the jejunum (*arrowhead*)

**Fig. 4:**
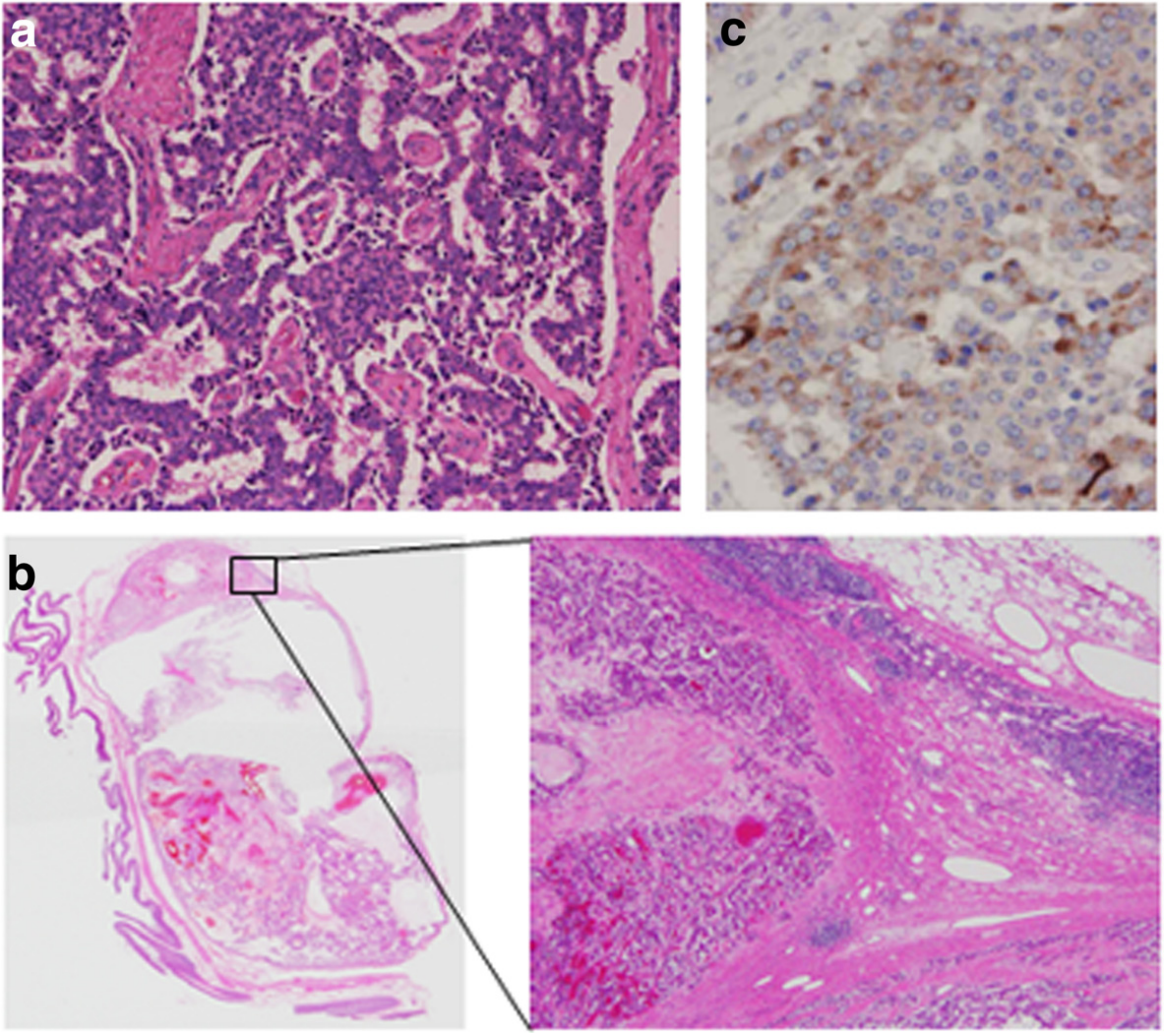
Microscopically, the tumor consists of a small-sized round cell proliferation with a solid nest pattern (**a**). The cyst preserved the structure of the lymph node and was the superior mesenteric lymph node metastasis of the tumor (**b**). Immunohistochemically, the resected specimen revealed that the tumor and lymph node metastases were positive for chromogranin A (**c**)

The patient was referred to our hospital for treatment of the NET. We performed a pylorus-preserving pancreaticoduodenectomy with regional lymph node dissection. The operative time was 497 min, and the volume of blood loss during the surgery was 1220 ml. The resected specimen was macroscopically a 9-mm white solid tumor (Fig. 3). Tumor cells confined to duodenal mucosal layer. There was a 52-mm cyst in the superior mesentery adjacent to the wall of the jejunum (Fig. 3). Microscopically, the tumor consisted of small-sized round cell proliferations with a solid nest pattern (Fig. 4a). The cyst preserved the structure of the lymph node and was the superior mesenteric lymph node metastasis of the tumor (Fig. 4b). We performed D2 lymph node dissection, and there was no metastatic lymph node except for the superior mesenteric lymph node. Immunohistochemically, the resected specimen revealed that the tumor and the lymph node metastasis were positive for chromogranin A (Fig. 4c) and CD56 and negative for synaptophysin. The Ki-67-labeling index of the tumor cells determined with MIB-1 was 2.0 %. The final diagnosis was sporadic non-functional NET G1 of the ampulla of Vater (pT1N1M0 stage IIIB). The patient was discharged 36 days after the operation. He has had no recurrence for 4 years after surgery. No further treatment was administered.

### Discussion

NETs of the ampulla of Vater are rare and difficult to diagnose [1, 2]. Jaundice (60 %) and abdominal pain (40 %) are the most frequent symptoms. Upper gastrointestinal bleeding is a rare presentation (<3 %) [1, 10]. In our case, the patient presented with abdominal pain and melena. On admission, upper gastrointestinal endoscopy revealed a bulging papilla with active bleeding. When we performed gastric endoscopy again 7 days later, we found a 10-mm mass of the ampulla of Vater with a superficial ulcer.

Diagnosis of NETs is established by histological and immunohistochemical analysis of endoscopic biopsy specimens [11]. NETs of the ampulla of Vater usually appear as submucosal masses that are small and spherical with a smooth surface and an intact duodenal mucosa. Consequently, superficial biopsies are negative and deeper biopsies are required for a diagnosis [12, 13]. In our present case, we could get the biopsy specimen from the mass because it had an ulcerated surface. Thus, the correct diagnosis of a NET was established preoperatively. However, we could not diagnose the cyst adjacent to the wall of the jejunum as a lymph node metastasis at the time of resection.

Previous reports have suggested that the biological behavior of ampullary NETs is distinct from that of duodenal NETs and they are more aggressive [14]. Randle and colleagues reported that ampullary NETs were larger, higher grade, and higher stage and had a higher rate of lymph node metastasis than duodenal NETs [15]. The incidence of lymph node metastases in patients with resected ampullary NETs and duodenal NETs was 72.9 and 48.4 %, respectively.

In the case of ampullary NETs, even in tumors smaller than 2 cm, a high percentage have lymph node metastases [14, 16–18]. Nikou et al. reported that lymph node metastases were found in two cases of ampullary NET with tumor sizes of 1.0 and 1.2 cm, respectively [19]. In the present case, the histologic examination revealed metastasis to distant lymph nodes despite the tumor only being 9 mm in diameter and within duodenal mucosal layer. These findings suggest that there is no correlation between tumor size and metastatic potential in ampullary NETs. We consider that an anatomical reason is one of the reasons why NETs at the ampulla of Vater have high incidence of metastasis. Ampullary carcinoma with perisphincteric or duodenal submucosal invasion showed more frequent lymph node metastasis and a greater tumor recurrence rate than tumor limited within the sphincter of Oddi muscle [20]. Moreover, the perisphincteric and duodenal submucosal space is relatively small and closer to the next layering of duodenal proper muscle and the pancreas. Thus, the malignant potential of perisphincteric and/or duodenal submucosal invasion may be greater than that of other gastrointestinal tract tumors. Moreover, previous studies have reported that lymph node metastasis is difficult to detect on preoperative imaging [18, 21]. Although some authors report the existence of lymph node metastasis of NETs is not correlated with patient prognosis, this could be due to metastasis to the liver or some other organ, which is an important prognostic factor for NETs. Therefore, radical resection with lymph node dissection is recommended as a treatment of ampullary NETs regardless of tumor size [1, 14, 18, 22, 23].

We performed a pylorus-preserving pancreaticoduodenectomy with regional lymph node resection and also resected a 52-mm lymphatic metastasis adjacent to the wall of the jejunum. Despite the lymph node involvement, the Ki-67-labeling index was low (2 %) and the patient has had no evidence of recurrence for 4 years after surgery.

Randle et al. indicated that tumor size was correlated with poor prognosis, but the presence of positive lymph nodes was not a predictive outcome in resected ampullary NETs [15]. Untch et al. reported that only tumor size and tumor grade were associated with recurrence [24]. Thus, we can perform radical resection and completely remove the tumor with good clinical outcomes.

## Conclusions

In conclusion, NETs of the ampulla of Vater, even very small tumors (<1 cm), may have aggressive metastatic potential. A radical resection with lymphadenectomy might be the appropriate treatment strategy for sporadic NETs of the ampulla of Vater.

## Consent

Written informed consent was obtained from the patient for publication of this case report and any accompanying images.

## Abbreviations

5-HIAA, 5-hydroxyindoleacetic acid; CEA, carcinoembryonic antigen; CT, computed tomography; NET, neuroendocrine tumor
